# President’s Address

**Published:** 1898-02

**Authors:** George H. Chance


### PRESIDENT'S ADDRESS.[1]

BY GEORGE H. CHANCE, D.D.S., M.D.

It is not my intention in this address to give you a long discourse on the rise and progress of modern dentistry, however profitable it might or might not be, nor to descant upon any particular phase of the science or art of our profession, but rather to throw out in a promiscuous sort of way a few suggestive thoughts for your careful consideration, and should some things be said as I proceed which may sound harsh in your ears, I trust you will pardon the plain language and consider not the letter but the spirit of the address, as well as make a proper application of the moral it is intended to convey.

It will doubtless be conceded by the reading and thinking members of this Association, and we should all be readers and thinkers, that while as individual dentists we may consider ourselves "up to date," yet as an organized body of professional workers we are very far behind our brethren in that respect in perhaps almost every other section of the country. Nor will it do in this age of steam and electricity to say, by way of excuse, that we are handicapped by distance from our *confrères;* that we have not the same facilities for study and original investigation which they possess; we cannot say that climatic influences have changed the character of our text-books, our journals, and other periodicals of the profession. What is it, then, which keeps us in the background of the progressive dental States? Have we no original ideas, no scientific theories of our own to bring to the light, or no inventive mechanical ability, from out of some of which Oregon may be able to set up one little "mile-stone" for the guidance of others on the road of progressive professional life? or are we ready to admit that the Oregon dentist knows no road to professional life; that he is a mere dental huckster, selling his wares for whatever he may be able to get from any shopping purchaser who may happen along? or will we consent that our professional brains have become so dull that we can gather no inspiration for an organized professional advance in this God-given land, with her snow-capped peaks, her ever fertile valleys, her mineral rocks and rills, her streams and wooded hills, her fruits and flowers, her schools and colleges, her churches and

---

[1] Read before the Oregon State Dental Association, October 14, 1897.

public libraries, her growing cities and thrifty farms, her manufactures and her commerce?

I think not; yet we cannot gainsay the fact that there exists an apathy and lethargy among Oregon dentists which ought not to be. What are the causes, and shall we try to find the answer? It is a trite saying that "God helps those who are willing to help themselves;" so with this quotation in our minds let each one ask himself not what the past year has done for him in the way of dollars and cents, for though a part, it is not all there is of dentistry, but rather what he himself has done or has attempted to do for the advancement of his profession. Whether or not he has been acting the part of a sponge, absorbing the thoughts, the ideas, and the work of his fellow-practitioners without giving out anything in return, or whether without undue squeezing he has himself contributed something of his own to the general fund of dental knowledge, or does he, like a stationary dead stick or stone, remain exactly in the same spot he occupied one year ago, except that he is covered with a little more moss, and is one year nearer the fossil stage of his existence?—these are pertinent questions which each one can answer to himself for himself, and if answered in accordance with the facts, I think we will be able to get at some of the causes of the trouble with which the average Oregon dentist is affected; then again, how little any of us would or could know if each one were left, without outside aid, to his own inner resources, and, therefore, how much we are indebted to others for our knowledge, our acquirements, and the manifold blessings we enjoy through their efforts in our behalf, and though each one's contribution may at the time seem but little in itself, it is in the adding of the mites of each that we reach the aggregate of all human knowledge.

Then do not forget that such meetings as these are for the general good, and with the proper effort on the part of each may be made profitable to all. Now, time, perhaps, will not permit us at this meeting to examine all the fruit-bearing trees of our professional knowledge and experience, but we can have, if we will, a few seed-thoughts that may be planted and co-operatively cultivated for future fruit-bearing. To generate these seed-thoughts may at first glance seem to be difficult, but look about you and then learn how little it takes to generate a seed-thought that may ultimately become a fruit-bearing tree if the mind be allowed to run in the proper channel. It was nothing but a falling apple that generated in the mind of a Newton the seed-thought of the law of gravitation, and naught but a flying kite which gave the seed-thought of the electric current to Franklin, and it was the boiling water in a teakettle which gave Watts the seed-thought of the invisible power of steam. And what was it but the pustule on the finger of a dairy-maid which gave the seed-thought of vaccination to a Jenner. Even the dental engine which dentists use, in its various makes and forms, is but the development of the seed-thought of a sheep-shearer; and so I might continue on, speaking of other seed-thoughts planted by past and present benefactors of the human race, which, by proper cultivation on the part of others, have become full fruit-bearing trees of knowledge and wisdom of which we are all partakers. But greatest and best of all these seed-thoughts was that of the "Fatherhood of God and the Brotherhood of man," planted near nineteen hundred years ago in a few faithful breasts by the Christ of Calvary, which by careful co-operative cultivation has become the splendid fruit-bearing tree of a Christian civilization, and which by its uplifting power through its gentle and benign influence has made possible the development of all other seed-thoughts planted and cultivated for the benefit of the human race; but without the all-important principle of co-operation these seed-thoughts, or, to change the figure, germs of progress, would have died at the moment of their birth, entirely changing the divine order of things from life and progress to retrogression, disintegration, inertia, and death.

I would have you, therefore, consider well this principle of co-operation if you desire to advance the standard of dental progress in the State of Oregon, for it is this same principle of co-operation in successfully developing the seed-thoughts of others which has made dentistry the profession that it is to-day, honored and respected by the intelligent and progressive of every community in the land. Then it becomes self-evident that in order to succeed in the fullest sense as individual dentists we must in the very nature of things co-operate with each other; but this principle of co-operation implies compatibility with and co-ordination of the several parts of the body before there can be full and perfect co-operation in any direction. Now, what are the facts so far as the great membership of the dental profession is concerned? Are not some of us afflicted with incompatibility? and does not the disease sometimes manifest itself in a lack of that true professional spirit which ought at all times to guide us in our actions towards each other? Do we not sometimes use unkind words when speaking of a brother practitioner, when kind words would be better for him and more creditable to us? Are we always careful to try to sooth the ruffled feelings of a scolding patient of a brother dentist for an alleged error or mistake attributed to said dentist, but through the sin of omission. Do we not sometimes rather aggravate the case against the brother, however unjust the charge, by not offering a kindly suggestion that the patient return to the offending dentist for explanation or correction of the alleged error.

Now, it has been demonstrated time and again that when such cases occur,—and they have occurred in other places besides Oregon,—that while all parties in interest must necessarily suffer, yet by the law of reflex action those who are afflicted with this form of incompatibility usually suffer the most, for the very good reason that our patrons are very much like ourselves, susceptible to outside impressions, be they good or bad.

We have doubtless all seen and have listened to the sounds emanating from the machine known as the phonograph. We also know that if discordant sounds are spoken into the machine that the same identical sounds will, under the proper stimulus, be discharged therefrom; and so, it seems to me, that where bad impressions are made by a dentist, either as regards himself, a brother dentist, or the profession at large, upon human phonographs in the form of dental patrons, such impressions are apt to become indelibly fixed in the minds of such persons, and perhaps can never be entirely eradicated, notwithstanding the utmost caution and the very best service one may be able to render them thereafter.

Before closing this address let me give you one or two snapshot pictures of some of the incompatibles. Here is one: a would-be patient, a gentleman, all smiles and affability, enters your dental office, and after being seated in the chair, proceeds to pour out his "tale of woe," how that Dr. —— has had charge of his mouth for some time past; but he is sorry to have it to say that Dr. —— has not given satisfaction; that you have been highly recommended to him as being one of the best, if not the best dentist in the city, and are very reasonable in your prices. He therefore desires to make an appointment with you at as early a date as you can give him, as he is a very busy man and his time is limited. Now, should any such individual visit your office, make the appointment, but first see that "wicked dentist" before operating, and ascertain if there is not an unpaid account standing against the aforesaid would-be gentleman patient; if so, govern yourself accordingly, as it may save you from a fit of profanity, labor wasted, and the loss of other and more valuable patients.

Here is one showing the " Uriah Heeps" of the profession, that class who are always "so very humble" that they never say anything to their patients that would sound naughty about a professional brother, but when canvassing the merits of another dentist who is not of their ilk, they merely shrug their shoulders, heave a deep sigh, and roll up their eyes in mock horror, to express their abhorrence at the wickedness of dentists in general, their own dear humble selves being the only exception. This one shows the "teeth without plates," dental vampire, who, like his prototype, mutilates and destroys in order the better to enable him to sell his false wares for whatever he can wrest from the pockets of his innocent victims, and this last one, from which most of the incompatibles originate, are a lot of cunning ignoramuses with little or no preliminary or special training, but depending entirely on their cunning to aid them in slipping by the sleepy sentinels who are supposed to watch and guard the gate-ways which open into the dental domain. Now, these are no fancy pictures, but true portraits taken from dental life as it exists, not only in Oregon but elsewhere in this country.

Permit me to impress upon the mind of each one present that as members of the Oregan State Dental Association we constitute an integral part of the great working force of American dentists, and as such integral part should act in harmony with the body of professional workers in other sections of the country, and this we may do by eliminating as far as possible from our own natures all that is incompatible with successful co-operative work, never taking part with the guerillas of the profession, but ever and always making legitimate war upon them until they are compelled to abandon their methods or be driven out from among us.

Thus and thus only can Oregon be brought into the front ranks of the progressive dental States, and thus only shall we be able to elevate ourselves, and by so doing secure the esteem of all the best elements in the profession, as well as gain the full confidence of the general public in us as professional men, while at the same time each co-worker will receive his full equivalent of such mutual co-operation.

